# Human ophthalmomyiasis caused by *Oestrus ovis* in Bulgaria: Case Report

**DOI:** 10.3389/fopht.2025.1689524

**Published:** 2025-11-25

**Authors:** Milena Atanasova Atanasova, Alexander Bozhidarov Blazhev, Elka Tzvetanova Milanova, Lidiya Plamenova Petrova

**Affiliations:** 1 Department of Anatomy, Histology, Cytology, and Biology, Section “Biology”, Medical University – Pleven, Pleven, Bulgaria; 2 Dr Stefan Cherkezov Regional Hospital, Veliko Tarnovo, Bulgaria

**Keywords:** ophthalmomyiasis, *Oestrus ovis*, parasitic eye infestation, Bulgaria, case report, myiasis

## Abstract

Ophthalmomyiasis is an uncommon parasitic infestation of the human eye caused by dipteran larvae, most frequently *Oestrus ovis*. It is rarely reported in temperate countries such as Bulgaria. We present a case of external ophthalmomyiasis in a 35-year-old urban resident, with no livestock exposure, caused by *O. ovis*. Larvae were morphologically identified after extraction. Approximately 30 motile larvae were mechanically removed over several days. Microscopy confirmed first-instar *O. ovis* larvae. Treatment included systemic and topical antibiotics, anti-inflammatory agents, and eye irrigation, resulting in complete recovery. This case of ophthalmomyiasis demonstrated that infestation can occur among urban residents without direct contact with livestock. Early recognition and mechanical removal are essential to prevent ocular damage.

## Highlights

- Ophthalmomyiasis, although rare in Bulgaria, occurs in both rural and urban populations.
- Awareness among ophthalmologists, general practitioners, and parasitologists is essential for early recognition and prevention of complications.
- Climate change and reduced vector control may increase future incidence.

## Introduction

Myiasis is an infestation caused by the larvae of insects belonging to the order Diptera. Members of this order deposit eggs or, in the case of larviparous species, live larvae onto the skin, within open wounds, or into various natural body orifices of vertebrates, including humans (1). The most important families capable of causing myiasis in various hosts are Oestridae, Calliphoridae, Muscidae, and Sarcophagidae (2, 3). Globally, the most common etiological agents of human myiasis are *Dermatobia hominis* and *Cordylobia anthropophaga*.

Depending on the localization of the larvae within the human body, myiasis can present as cutaneous, nasopharyngeal, ophthalmomyiasis, intestinal, or urogenital forms (4). The clinical case presented herein describes ophthalmomyiasis caused by *Oestrus ovis* larvae.

The family Oestridae comprises 28 genera and 151 species causing myiasis. Morphologically, they are classified into four subfamilies: Oestrinae, Gasterophilinae, Hypodermatinae, and Cuterebrinae (4, 5). Oestrinae includes four genera, among which the genus *Oestrus* contains *O. ovis*, the sheep nasal botfly, which infests sheep, goats, and occasionally humans (6, 7). These flies typically deposit eggs or larvae on the skin or near body openings and, in some cases, directly into or around the host’s eyes.

Ophthalmomyiasis is the infestation of the eye by dipteran larvae. It is categorized into external (involving the eyelids and conjunctiva), internal (with penetration of larvae into the globe), and orbital (affecting the optic nerve and surrounding orbital tissues).

External ophthalmomyiasis usually presents with an acute onset, often within hours of contact with the fly. Symptoms include redness, burning, tearing, swelling, and a foreign body sensation in the eye. A characteristic complaint is the feeling of movement between the globe and the conjunctiva. Live, motile larvae can often be visualized by slit-lamp examination. Mild corneal involvement (keratitis) has been reported (8).

If not diagnosed in time, larvae may penetrate ocular structures, including the anterior chamber, vitreous body, and retina, and cause internal ophthalmomyiasis (9). This rarer but more severe form may present with floaters, photopsia, ocular pain, decreased visual acuity, vitreous haemorrhage, and retinal degeneration.

Orbital involvement damages the optic nerve and surrounding tissues, requiring aggressive treatment. If left untreated, it can lead to severe complications, including vision loss (10).

The most frequent cause of external ophthalmomyiasis in humans is *O. ovis*, particularly among individuals living or working in proximity to sheep and goats. Other species capable of causing both external and internal ophthalmomyiasis include *D. hominis*, *Chrysomya bezziana*, *Hypoderma tarandi*, and *Musca domestica* (11).

In Bulgaria, *O. ovis* is present throughout the country. According to an online publication from 2015 (12), its prevalence among sheep flocks ranges from 70% to 100%, as reported in surveys in Sardinia (13). Warm, dry weather favours parasite development. In Bulgaria and the Balkan Peninsula, *O. ovis* is larviparous (14) and produces two generations per year: one from early June to late July and the other from late August to October–November (12). Adult flies are grey-brown, 10–12 mm in body length, with sparse hair, small black spots on the thorax, and dull yellow head and legs. The mouthparts are vestigial, as adults do not feed during their approximately 2-week lifespan (14). After mating, the males die, while the females remain immobile for 10–20 days on walls, fences, and cracks where larval development occurs.

Females deposit between 8 and 40 live larvae into or around the nostrils of sheep and goats. Over their lifetime, each female releases approximately 350–500 larvae. Once inside the nasal passages, the larvae attach to the mucosa and migrate slowly toward the frontal sinuses and horn cavities, where they remain for 8–9 months throughout autumn and winter. During this time, they moult from first (L1) to second (L2) and third (L3) instars, reaching lengths of 20–30 mm. In spring, mature L3 larvae exit the sinuses and pupate, and after 38–65 days, they emerge as adults, restarting the cycle (13).

Usually, *O. ovis* larvae parasitize the nasal cavities, frontal sinuses, and, occasionally, the maxillary sinuses of sheep and goats (6), but have also been reported in other ruminants, including llamas (15).

**Figure 1:**
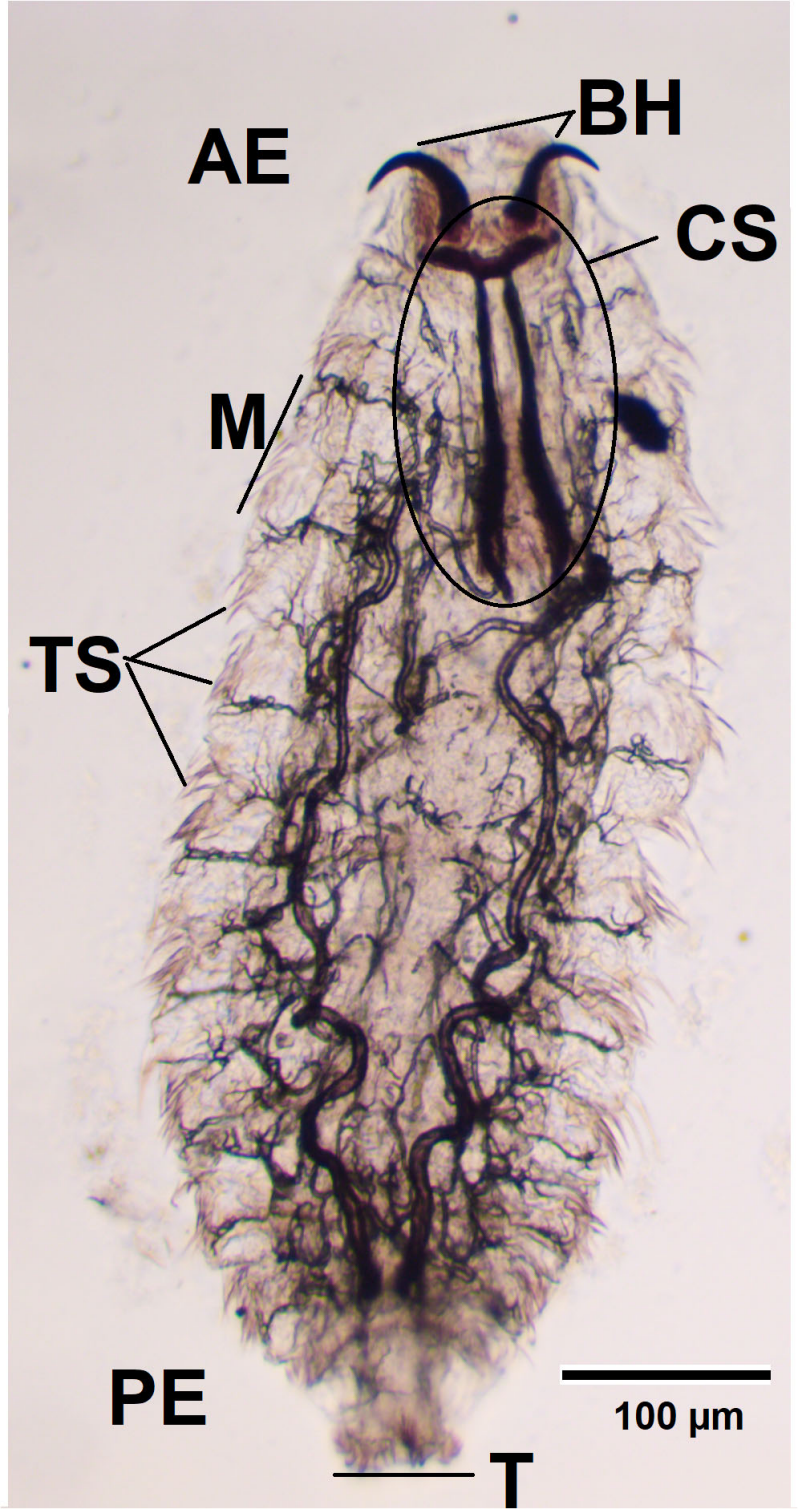
Larva fixed in formalin, observed using a light microscope (×100). АЕ, anterior end; BH, buccal hooks; CS, cephalopharyngeal skeleton; M, metamere; TS, tufts of spines; T, tubercles; PE, posterior end.

First-instar larvae (L1) are translucent white, fusiform, dorsoventrally flattened, and elongated, measuring approximately 1–5 mm in length. The body consists of 11 segmented metameres, a relatively large cephalothoracic skeleton (**Figure 1**), and a pair of strongly curved oral hooks (**Figure 1**, BH) visible under a microscope. The hooks are small and serve to facilitate mucosal penetration and attachment. On the ventral side of the anterior segments, two to three complete rows of spines are present. The posterior end bears 22–25 terminal hooks arranged in two pads, with small dorsal spines on the third segment. A pair of dark yellow spiracles is visible posteriorly. L1 larvae actively migrate through the nasal mucosa.

Second-instar larvae (L2) are whitish to pale yellow, 9–15 mm long, with several small dorsal spines on the second segment and fine spines in the median area of the post-anal swelling. The ventral segments carry multiple rows of backwards-pointing comb-like spines, aiding in attachment and locomotion. At this stage, the posterior spiracles are well-developed, and the oral hooks are more prominent (16).

Third-instar larvae (L3) measure 14–27 mm, initially white to yellowish in colour, and gradually darken to light brown with a transverse reddish to black dorsal band in mature specimens. Numerous irregular small denticles are found dorsally on the second segment, while the subsequent segment is smooth. The ventral surfaces of all segments have regularly arranged spine rows, except the third segment, where they are irregular. The pre-anal swelling is spineless, whereas the post-anal swelling carries a few spines. Posterior spiracles turn black at this stage (17).

## Case report

A 35-year-old man presented to the ophthalmologist after an incident in which a small object, seemingly a fly, struck his eye while he was walking past a meadow. Following the incident, he experienced ocular irritation, pain, and a sensation of movement inside the eye. The initial examination revealed conjunctival hyperemia and corneal defects, but no foreign bodies were detected.

**Figure 2:**
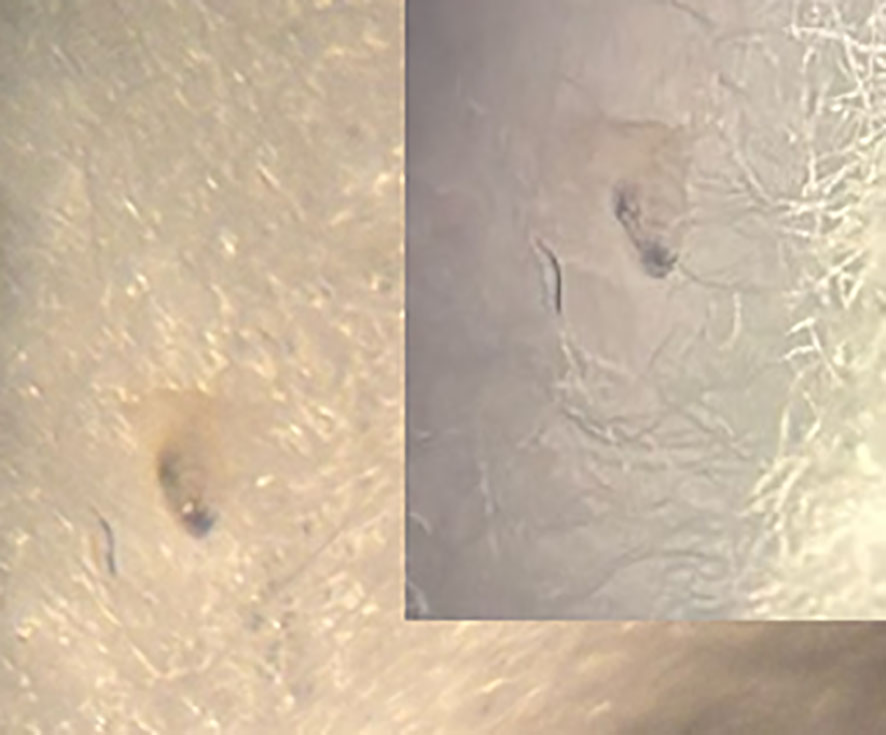
First-instar *Oestrus ovis* larva extracted from the conjunctiva showing translucent bodies and dark anterior ends.

The patient was immediately admitted to Dr Stefan Cherkezov Regional Hospital in Veliko Tarnovo. On subsequent examinations for persistent complaints of a moving foreign body sensation in his eye, several small, rapidly motile, translucent objects were detected. It was then presumed that, upon striking the patient’s eye, the suspected fly had deposited its larvae. Over several days, the attending ophthalmologist removed about 30 such objects. On the first day, these were less than 1 mm in size (**Figure 2**), barely visible to the naked eye (no microscopic measurements were performed). By the second day, they had visibly increased in size. They could not be removed by irrigation or swabbing and were extracted only with forceps. The second-day specimens were translucent, with darkened areas at one end, and were still no more than 1 mm in size.

Clinically, it was first assumed that these were Diptera larvae. Over the following days, the larvae grew to several millimetres. On the second day, three of the extracted specimens were preserved in formalin and sent to the Department of Anatomy, Histology, Cytology, and Biology at the Medical University – Pleven for microscopic identification. The specimens were examined using an Optica light microscope (Optica, Italy) at ×40 and ×100 magnification and mounted in Eukitt^®^ Quick-hardening medium (Sigma-Aldrich, St. Louis, Missouri (MO), USA; cat. no. 03989) for permanent slides.

Macroscopically, the larvae were milky white and less than 1 mm in length (**Figure 2**). To measure the length and width of the larvae, we used an eyepiece micrometre and found that they were 0.7–1 mm long and 0.26–0.35 mm wide. Microscopically, they were fusiform and segmented (11 metameres; **Figure 1**, M) with tufts of spines between segments (**Figure 1**, TS). A pair of dark brown buccal hooks was located anteriorly (**Figure 1**, BH), connected to a cephalopharyngeal skeleton (**Figure 1**, CS). Comparison of these microscopic features (presence of morphological characteristics such as the cephalopharyngeal skeleton and anterior hooks) and their size with published descriptions (18, 19) identified the specimens as first-instar larvae of *O. ovis*.

Treatment included intravenous administration of ceftriaxone and gentamicin, non-steroidal anti-inflammatory agents, and topical ocular therapy with levofloxacin, netilmicin, ozonated oil drops (Ozodrop, FB Vision, Ascoli Piceno, Italy), tropicamide, and ofloxacin ointment. The eye was irrigated with saline and diluted povidone-iodine solution. Treatment with intravenous antibiotics was administered due to the pronounced inflammatory process in the eye—mixed infection, purulent discharge, multiple superficial and deep defects, and corneal edema—leading to a significant reduction in vision. In addition, there was hyperemia, a smooth iris structure, and a narrow pupil that did not respond to medical mydriasis. The described eye status indicated a secondary bacterial infection and spread of the inflammatory response to the anterior chamber of the eye, requiring antibiotic therapy.

Treatment was carried out in the hospital for 5 days, with two examinations per day using light biomicroscopy. During the first 3 days, parasites were removed, and by the fourth day, all parasites had been completely eliminated.

During hospital treatment, visual acuity was reduced to 0.3 (30%). On discharge, the condition of the eye was described as follows: the infection had completely subsided, the corneal defects had almost completely epithelialized, the edema had been resorbed, the iris had regained its structure, and the pupil was in maximum medicated mydriasis. Visual acuity was completely restored. After discharge, three follow-up examinations were performed within 1 month. The eye was completely healthy.

## Discussion

Myiasis was first described by Hope in 1840 (20), and the first reported case of human ophthalmomyiasis caused by *D. hominis* (“the human botfly”) was published by Keyt in 1900 (21).

*O. ovis*, the sheep nasal botfly, is primarily an obligate parasite of sheep and goats, with larvae usually inhabiting the nasal passages and sinuses of these hosts. However, it can occasionally cause ophthalmomyiasis or, less frequently, nasopharyngeal myiasis in humans. Worldwide, approximately 70% of human ophthalmomyiasis cases are attributed to *O. ovis* (Martinez-Rojano, 2023).

Human ophthalmomyiasis and nasopharyngeal myiasis caused by *O. ovis* have been documented globally—in France (18, 22), Nepal (23), India (24, 25), Egypt (26), Turkey (27, 28), Saudi Arabia (29), Mexico (30), Bosnia (31), Peru (32), and many other countries.

The present case is only the second reported in Bulgaria. The first, described by Velev and Mikov (33), involved combined ophthalmomyiasis and nasopharyngeal myiasis. Humans are accidental hosts, and the primary risk groups include individuals raising sheep or goats and those living near livestock facilities. Most cases have a history of contact with farm animals or rural exposure. Additional risk factors include poor hygiene, periorbital wounds, and specific comorbidities such as obesity, diabetes, or necrotic/malignant lesions near the orbit, which may facilitate larval invasion (10).

Recent years have seen an increase in reported cases among urban residents without occupational or rural exposure (8). Our patient had a good social status, no occupation-related risk factors, and no close contact with livestock, indicating that ophthalmomyiasis can occur in individuals across all social and occupational backgrounds.

|The diagnosis of external ophthalmomyiasis is established by slit-lamp visualization of larvae on the conjunctiva or cornea. In suspected internal ophthalmomyiasis, fundus photography, ocular ultrasonography, or MRI may be indicated. Larval identification under microscopy remains challenging due to morphological similarities among *Oestrus* species (34).

Treatment consists of mechanical removal with sterile forceps under local anaesthesia. Topical anaesthetic agents may paralyse larvae, facilitating extraction (35, 36). *O. ovis* larvae are photophobic, tending to migrate toward shaded areas (37), so ectropionization is essential during examination and removal (26). The larvae’s buccal hooks anchor firmly to conjunctival and nasal mucosa, making irrigation alone insufficient (31). In our case, saline irrigation and swabbing were ineffective, requiring forceps extraction.

Post-extraction management includes topical antibiotics (e.g., tobramycin or moxifloxacin) and anti-inflammatory drops. Ivermectin, administered orally or topically, has been used with variable success but is considered to facilitate removal and reduce the risk of recurrence (38–40). A follow-up examination within 24–48 hours is recommended to ensure complete removal, as even remnants of larvae may cause persistent inflammation or secondary infection.

Preventive measures include avoiding direct contact with livestock, particularly in endemic rural areas, wearing protective eyewear during outdoor work, maintaining good personal hygiene, and implementing fly control programs.

## Conclusion

Ophthalmomyiasis and other parasitic ocular infestations in humans are exceptionally rare in Bulgaria but do occur, regardless of patient age or sex. Importantly, the intermediate vector is not always a domestic animal. Some clinical cases result from accidental contact with flies that enter through open windows or from outdoor exposure.

Only one other Bulgarian case of ophthalmomyiasis—combined with nasopharyngeal myiasis—has been reported (33), diagnosed several days after infestation. Our report, together with the earlier one, confirms the presence of this parasitosis in Bulgaria despite its rarity. Even in a predominantly urban, non-tropical country, ophthalmomyiasis should be considered in the differential diagnosis of conjunctivitis-like symptoms, particularly against the background of a history of ocular foreign body entry.

Climate change and reduced insect population control may increase the likelihood of future cases. Rapid diagnosis and intervention are essential to prevent complications and vision loss. The gold standard of treatment remains prompt mechanical removal of larvae combined with antibiotic and local therapy (41).

Awareness should be raised not only among ophthalmologists but also among general practitioners, pathologists, parasitologists, and infectious disease specialists to facilitate timely recognition and management.

Although ophthalmomyiasis is generally self-limiting and usually resolves rapidly after treatment, it can lead to severe complications if diagnosis or treatment is delayed. Thus, it must be included among provisional diagnoses in patients presenting with acute conjunctivitis symptoms and unilateral foreign body sensation. Increased migration to rural areas, including among individuals seeking isolation or remote living, may lead to more frequent encounters with such cases in the future.
